# Change in Intraocular Pressure During Point-of-Care Ultrasound

**DOI:** 10.5811/westjem.2015.1.24150

**Published:** 2015-03-06

**Authors:** Cameron Berg, Stephanie J. Doniger, Brita Zaia, Sarah R. Williams

**Affiliations:** * North Memorial Health Care, Department of Emergency Medicine, Robbinsdale, Minnesota; † University of California, San Francisco Benioff Children’s Hospital Oakland, Division of Emergency Medicine, Oakland, California; ‡ Kaiser Permanente, San Francisco Medical Center, Department of Emergency Medicine, Palo Alto, California; § Stanford University Medical Center, Division of Emergency Medicine, Department of Surgery, Palo Alto, California

## Abstract

**Introduction:**

Point-of-care ocular ultrasound (US) is a valuable tool for the evaluation of traumatic ocular injuries. Conventionally, any maneuver that may increase intraocular pressure (IOP) is relatively contraindicated in the setting of globe rupture. Some authors have cautioned against the use of US in these scenarios because of a theoretical concern that an US examination may cause or exacerbate the extrusion of intraocular contents. This study set out to investigate whether ocular US affects IOP. The secondary objective was to validate the intraocular pressure measurements obtained with the Diaton® as compared with standard applanation techniques (the Tono-Pen®).

**Methods:**

We enrolled a convenience sample of healthy adult volunteers. We obtained the baseline IOP for each patient by using a transpalpebral tonometer. Ocular US was then performed on each subject using a high-frequency linear array transducer, and a second IOP was obtained during the US examination. A third IOP measurement was obtained following the completion of the US examination. To validate transpalpebral measurement, a subset of subjects also underwent traditional transcorneal applanation tonometry prior to the US examination as a baseline measurement. In a subset of 10 patients, we obtained baseline pre-ultrasound IOP measurements with the Diaton® and Tono-Pen®, and then compared them.

**Results:**

The study included 40 subjects. IOP values during ocular US examination were slightly greater than baseline (average +1.8mmHg, p=0.01). Post-US examination IOP values were not significantly different than baseline (average −0.15mmHg, p=0.42). In a subset of 10 subjects, IOP values were not significantly different between transpalpebral and transcorneal tonometry (average +0.03mmHg, p=0.07).

**Conclusion:**

In healthy volunteer subjects, point-of-care ocular US causes a small and transient increase in IOP. We also showed no difference between the Diaton® and Tono-Pen® methods of IOP measurement. Overall, the resulting change in IOP with US transducer placement is considerably less than the mean diurnal variation in healthy subjects, or pressure generated by physical examination, and is therefore unlikely to be clinically significant. However, it is important to take caution when performing ocular ultrasound, since it is unclear what the change in IOP would be in patients with ocular trauma.

## INTRODUCTION

Point-of-care ultrasound (US) was introduced into emergency medicine in the 1980s. Portable ultrasound machines are now readily available in many emergency departments (ED), and physicians are becoming increasingly facile with a variety of diagnostic and procedural applications. Ocular US was recently added to the American College of Emergency Physician’s (ACEP) core applications,^1^ and is recommended for the evaluation of retinal detachment, vitreous hemorrhage, lens dislocations/disruption, intraocular foreign bodies, retrobulbar hemorrhage, globe rupture, and as an indirect measurement of intracranial pressure.^1^–^4^ Point-of-care ultrasound can be performed on a closed eyelid, readily penetrating soft tissue swelling, while causing minimal discomfort to the patient.

Patients with potential globe rupture represent a particular diagnostic dilemma for emergency physicians. The rapid diagnosis of a globe rupture is imperative, since outcomes depend upon timely operative intervention. However, the diagnosis using clinical examination alone can be very challenging. Associated swelling of the eyelid often makes physical examination of the eye difficult, and attempts to manually open the eyelid in these situations may apply unwanted pressure on the globe despite careful precautions. Traditional imaging modalities used to aid in globe rupture diagnosis include radiographs, magnetic resonance imaging (MRI), and computed tomography (CT), but all are limited for various reasons. Conventional radiographs are able to evaluate for major bony injury or metallic foreign bodies, but with an exceedingly low sensitivity. MRI is frequently unavailable for emergent cases, is time-consuming, and is contraindicated in the setting of potential metallic foreign bodies. A thin-section helical CT is often considered to be the optimal study, but it also requires patient stabilization, transport outside of the ED, and exposure to ionizing radiation. More recently, point-of-care US emerged as an alternative modality in the setting of acute ocular trauma. However, controversy remains regarding the safety and theoretical increase in intraocular pressure (IOP) with placement of the US transducer onto the closed eyelid; the theoretical concern is that an increased IOP from external pressure may cause an extrusion of intraocular contents.^5^ No study to date has specifically investigated the impact of US on IOP in human subjects.

To evaluate whether the US transducer causes an increase in IOP, the IOP must be measured simultaneously while performing ocular US. A particular challenge arises in the method by which IOP can effectively be measured simultaneously with transducer placement. Tonometry, or the measurement of IOP, has been traditionally measured by applanation devices, which require a compliant patient with an open eyelid. The most common applanation device, the Tono-Pen®, involves compression of a small sensor against the surface of the patient’s cornea; an internal microprocessor approximates the IOP from the resistance and deformation of the cornea. Recently the Diaton®, a new type of non-corneal contact tonometer, has been developed, in which pressure can be measured on a partially closed eyelid. The Diaton® has a detector that measures the force necessary to deform the underlying sclera, and digital references are used to approximate IOP. Several studies have compared IOP measurements from this device. Results have been rather controversial: some studies have found concordant results with conventional tonometry,^6^–^8^ while others show limited accuracy with wide confidence intervals.^9^–^12^

We sought to determine the effect of point-of-care US on IOP in healthy human subjects. Our hypothesis is that the careful placement of an ultrasound transducer on the closed eyelid with a copious amount of gel, creates a minimal increase in intraocular pressure. Our secondary objective was to compare the Diaton® to Tono-Pen® techniques to validate the accuracy of measurements.

## METHODS

This is an institutional review board-approved prospective observational study performed on a convenience sample of 40 healthy volunteers. The study was conducted at a tertiary care university medical center. The subjects were healthy volunteers recruited from the emergency medicine residency program and affiliated administrative offices; there were no incentives or payments made to volunteers. Informed written consent was obtained from each subject. We excluded people with known pre-existing ophthalmologic pathology (i.e. glaucoma, retinal detachment, lens dislocation), history of eye trauma, allergy to systemic or local anesthetic agents, and the inability to provide informed written consent.

Four study investigators performed the ocular US examinations and IOP measurements: three emergency medicine residents and one emergency ultrasound fellow. Each operator was credentialed specifically in ocular US prior to participation in the study; this credentialing process followed ACEP guidelines^1^ and the guidelines were approved by the participating academic department and ultrasound division. The operator who performed the ocular US examination on each subject was blinded to that subject’s IOP results measured before, during, and after the US examination. Both thermal ultrasound images and 6-second video clips were obtained and recorded. US fellowship-trained faculty directors later reviewed the saved clips in order to ensure the adequacy and uniformity of ultrasound images.

**Figure 1:**
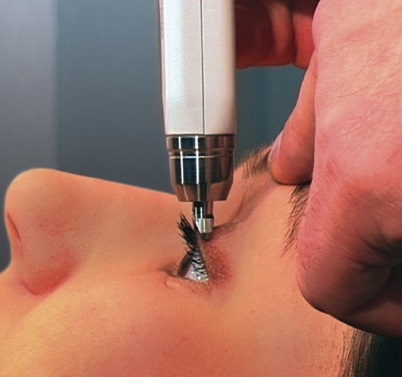
Diaton® transpalpebral tonometer. Photograph depicting the manufacturer’s recommendation for placement of the tonometer on a partially open eyelid.

**Figure 2:**
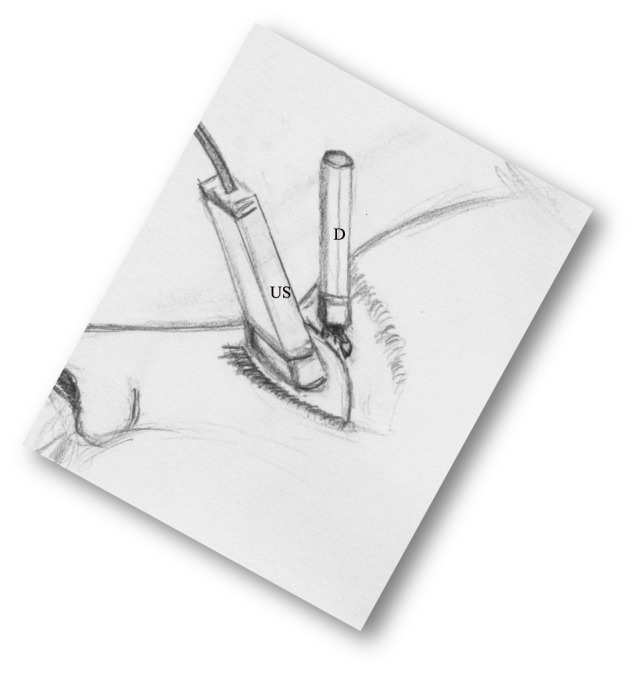
Graphic representation of the modified apparatus setup. The study subject is in a seated upright position, with a closed eyelid. The ultrasound transducer (US) is placed over the eyelid and the Diaton® transpalpebral tonometer (D) placed superior to the transducer.

This was a pilot study using a convenience sample. Once identified and consented for enrollment, each subject was assigned a study identification number and the researchers recorded data as it was collected. Each subject underwent three intraocular pressure measurements: prior to ultrasound, during ultrasound, and following ultrasound. All IOP measurements were performed in triplicate, and the mean values were recorded. A baseline IOP measurement first was obtained and recorded for either the right or left eye of each subject. This was recorded as the “pre-US” IOP. We used the Diaton® transpalpebral tonometer (BiCom Inc. Long Beach, NY). The manufacturer’s instructions recommend performing measurement on a person in the seated upright position, with a partially open eye by placing the device along the tarsal plate of the superior eyelid. We amended the technique, performing the measurement on a closed eyelid. The subject was placed in a seated position with eyes closed; the tonometer was centered on the eyelid at the superior aspect of the globe and angled perpendicular to ground level (Figure 1). After obtaining the baseline IOP measurements, ocular US was performed on the same eye. A 10-5MHz linear transducer (M-Turbo, Sonosite Inc.) was used and US gel was liberally applied to the transducer surface. The examiner’s hand was braced against the subject’s maxilla to minimize transmitted force. The transducer was placed directly on the closed eyelid, and the optimal transverse US image was obtained. While maintaining this image, a second researcher simultaneously performed transpalpebral tonometry by placing the Diaton® superior to the US transducer (Figure 2). This second IOP measurement was recorded as the “intra-US” IOP. Lastly, the US transducer was removed from the subject’s eyelid and the final transpalpebral IOP was measured and recorded as the “post-US” IOP.

To evaluate the accuracy of our amended transpalpebral technique, we randomly selected 10 of the study subjects to undergo an additional baseline, “pre-US” IOP measurement using the standard applanation tonometer, the Tono-Pen®. For the baseline applanation tonometry subjects, we administered two drops of sterile ophthalmic topical anesthetic prior to examination.

We entered data into a spreadsheet using Microsoft Excel (Redmond, WA). Paired t-test analyses were performed using SOFA Statistics (www.sofastatistics.com).

## RESULTS

Forty potential subjects were approached for enrollment, and all agreed to participate and completed the study. We studied 52.5% female and 47.5% male subjects, with a mean age of 31.8 years of age (range 19–59 years); 22.5% of the subjects were contact lens wearers. Contact lenses were all removed prior to study participation.

**Figure 3:**
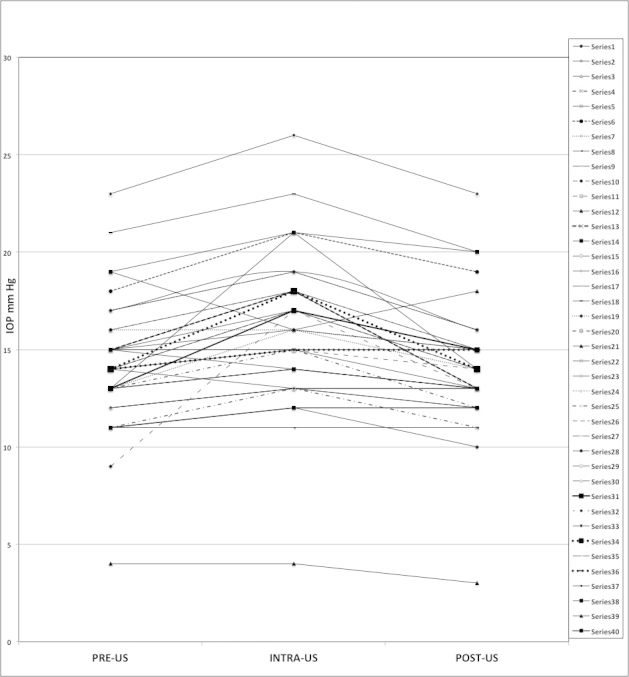
Graphic representation of the results. Each patient’s values of IOP (mmHg) are plotted to show the “Pre-US” IOP, “Intra-US” IOP, and “Post-US” IOP values. *US,* ultrasound; *IOP*, intraocular pressure

We calculated mean IOP values for each phase of measurement. “Pre-US” IOP values were 14.28mmHg (95% CI [13.26–15.29]). “Intra-US” IOP values were slightly greater than “pre-US” at 16.08mmHg (95% CI [14.92–17.23]). “Post-US” IOP values were 14.13mmHg (95% CI [13.14–15.11]). Comparing “pre-US” to “post-US” values, the difference of −0.15mmHg (range −3 to +3mmHg) and was not statistically significant (p=0.42). When comparing “pre-US” to “intra-US” IOP values, there was a statistically significant change of +1.8mmHg (range −1 to +7mmHg, p=0.01). The distribution of IOP values for each patient (Figure 3) shows that the maximum increase in IOP was 8mmHg, while for the majority of patients the increase was only 1mmHg (n=12) or 2mmHg (n=10). Overall, 87.5% of patients had an increase of 3mmHg or less.

We compared IOP values between the different methods of tonometry: the traditional Tono-Pen® transcorneal tonometry with the Diaton® tanspalpebral tonometry the difference between the techniques was not statistically significant (+0.03mmHg, range −3 to +4mmHg, p=0.07).

## DISCUSSION

Previous studies have established the accuracy and utility of point-of-care ocular US examinations.^13^,^14^ Blaivas et al.^13^ prospectively studied 61 patients: 26 were found to have ocular pathology, three with penetrating globe injuries. Overall, point-of-care ocular US was shown to be accurate, with a sensitivity of 100% (95% CI [94%–100%]) and a specificity of 97.2% (95% CI [89%–99%]) in identifying ocular pathology. Following a brief training, Chandra et al.^15^ showed that emergency physicians were able to identify ocular rupture in porcine eyes with a sensitivity of 79% and specificity of 51%. In this porcine model, IOP was only increased by 5% with the ultrasound transducer placement. Since there is a paucity of literature regarding the potential increase in IOP with US transducer placement, it is generally advised that caution should be exercised when performing ocular US in the setting of trauma. In these settings, there is particular concern that additional pressure on the orbit may potentially worsen a traumatized or already ruptured globe. We are not aware of any previous studies that have investigated the potential adverse effects of ocular ultrasonography and its effect on IOP in human subjects.

We designed this study to determine the change in IOP during point-of-care ocular US. Our results demonstrate that there is a small and transient increase in IOP during ocular US, with a mean of 1.8mmHg. The magnitude of this change, however, is unlikely to be clinically significant, largely since this is less than the mean diurnal variation in IOP, and less than the IOP generated with physical examination and eyelid speculum. De Venecia et al.^16^ found a mean daily IOP variation of 5.9mmHg in healthy subjects. In addition, the IOP has been shown to increase with maneuvers often performed in order to open and examine swollen, traumatized eyes. Gandhi et al.^17^ showed that when the examiner held eyelids open while subjects attempted forced eyelid closure, there was an increase in IOP of 1.9 +/− 2.7mmHg (p=0.0002, paired t-test range, −2 to 9mmHg) as measured with the Tono-Pen®. In certain situations, a lid retractor, or eyelid speculum may be used to visualize a swollen, traumatized eye. Epley et al.^18^ showed that in pediatric patients, an eyelid speculum increased IOP on average of 4 mm Hg, which was statistically significant for each eye individually as well as aggregately. This finding was also higher than the pressure generated by the US transducer in this study.

It is unclear at what point increased IOP represents a dangerous level, in which intraocular contents would be extruded and/or ocular pathology exacerbated. Vachon et al.^19^ described the extremely rare extrusion of intraocular contents despite average increases of 7mmHg when succinylcholine was administered. In addition, Zauberman et al.^20^ studied a rabbit model and showed that extrusion occurred most consistently with pressures over 30mmHg. Further, the FDA has established a criterion for elevated IOP in its evaluation of prospective medications. Their published standard is a change from baseline of ≥10mmHg, which represents a potentially dangerous increase in IOP.^21^ Overall, though further study is necessary in populations with ocular pathology, we believe that clinicians should be reassured that point-of-care ocular US examination causes a very small elevation in IOP when performed carefully as described above. This knowledge has the potential to allow emergency physicians to safely use ocular US in the ED to diagnose ocular pathology rapidly, non-invasively, and accurately.

Finally, we used transpalpebral tonometry, a novel technique that permitted us to measure IOP in real time during the ocular US exam while subjects’ eyes were closed, and without direct corneal contact. Traditional tonometry cannot be performed simultaneously while performing an ocular ultrasound since it requires an open eye. While several studies have previously compared the Diaton® to traditional tonometry, there have been conflicting results,^9^–^11^ and therefore its accuracy has been questioned. Nakakura et al.^12^ compared IOP measurements with four different methods of tonometry; the Diaton® did not correlate well, resulting in a wide bias range with wide confidence intervals. However, we found no significant difference between transpalpebral tonometry and transcorneal tonometry pressures in our comparative samples, supporting the validity of this novel technique.

## LIMITATIONS

One of the major limitations is that this study only included normal healthy subjects, which resulted in a small increase in intraocular pressure. Our findings may have been different had we studied subjects with acute or chronic ocular pathology. It is unclear whether this increase would adversely affect an individual with ocular pathology such as globe rupture, orbital foreign body, and retrobulbar hematoma. Therefore, further studies need to be performed in patients with ocular pathology.

Other factors may have influenced the IOP measurements. While it has been described that a clear plastic barrier shield may be used to minimize an increase in IOP, it was not used in this study. It is unclear whether this would have affected the results of the IOP changes. In addition, the study investigators were not blinded to the study objectives and ultrasound images. This could have introduced an inherent bias to the results. However, this is unlikely as practitioners concerned about globe injury would be cognizant of the pressure they are applying with US.

With regards to our secondary objectives, there are some limitations in comparing the Diaton® with traditional tonometry. We used a modified technique, different from that which is recommended by the Diaton® manufacturer. The manufacturer specifies a partially closed eyelid, whereas we had a fully closed eyelid, in order to be able to perform the US. Subjects were also in a sitting rather than in a supine position. Prior studies have shown that IOP is higher in the supine position,^22^ but it is only an increase in 1mmHg, which is unlikely to be clinically significant.^23^ It is also unclear whether measurements would correlate as well, in the setting of a severely swollen eyelid. Further studies would need to be performed to evaluate this.

## CONCLUSION

Point-of-care ocular ultrasonography causes a small and transient increase in IOP in healthy volunteers. We also showed no difference between the Diaton® and Tono-Pen® methods of IOP measurement. Overall, the resulting change in IOP with US transducer placement is considerably less than the mean diurnal variation in healthy subjects, or pressure generated by physical examination, and is therefore unlikely to be clinically significant. However, it is important to take caution when performing ocular ultrasound, since it is unclear what the change in IOP would be in patients with ocular trauma.
